# Origins and spread of novel genetic variants of sulfadoxine–pyrimethamine resistance in *Plasmodium falciparum* isolates in Indonesia

**DOI:** 10.1186/s12936-018-2597-6

**Published:** 2018-12-17

**Authors:** Sukmawati Basuki, Fitriah, Petronella M. Risamasu, Kasmijati, Pancawati Ariami, Sugeng Riyanto, Ari Hidayat, Dewi Susilowati, Iskandar, Budi Armika, Budiono, Yoes P. Dachlan, Hiroji Kanbara, Haruki Uemura

**Affiliations:** 1 Department of Medical Parasitology, Faculty of Medicine, Universitas Airlangga, Surabaya, Indonesia; 2 Malaria Study Group/Laboratory of Malaria, Institute of Tropical Disease, Universitas Airlangga, Surabaya, Indonesia; 3 Disease Control of Jayapura District Department of Health, Jayapura, Papua Province Indonesia; 4 UPTD, Puskesmas Kuala Pembuang, Seruyan District, Middle Kalimantan Province Indonesia; 5 Poltekkes Mataram, Kota Mataram, Lombok, West Nusa Tenggara Province Indonesia; 6 Banjar District Department of Health, Martapura, Banjar, South Kalimantan Province Indonesia; 7 Arifin Achmad Hospital, Pekanbaru, Riau Province Indonesia; 8 Faculty of Public Health, Universitas Gorontalo, Biyonga, Limboto, Gorontalo Province Indonesia; 9 Puskesmas Utan Rhee, Utan Rhee sub-district, Sumbawa, West Nusa Tenggara Province Indonesia; 10 West Nusa Tenggara Provincial Department of Health, Kota Mataram, Lombok, West Nusa Tenggara Province Indonesia; 11 Department of Public Health and Preventive Medicine, Faculty of Medicine, Universitas Airlangga, Surabaya, Indonesia; 12 Department of Protozoology, Institute of Tropical Medicine, Nagasaki University, Nagasaki, Japan

**Keywords:** *Plasmodium falciparum*, *Pfdhfr*, *Pfdhps*, Mutation, Polymorphism, Indonesia

## Abstract

**Background:**

While malaria incidence in Indonesia has decreased threefold in the last decade, more than 200,000 cases were reported in 2016. Different endemicity of *Plasmodium falciparum* malaria among several islands in Indonesia has been recognized and two unique mutations of *P. falciparum* dihydropteroate synthase (*pfdhps*) affecting sulfadoxine–pyrimethamine (SP) resistance were detected from the research of SP efficiency and genotype analysis in South Kalimantan. In this study, geographical distribution and origin of these *pfdhps* K540**T** and I588**F** mutations were analysed.

**Methods:**

Malaria parasites DNA from several endemic areas in Indonesia; Sumatera, Java, Kalimantan, Lombok, Sumbawa, Timor, Sulawesi, and Papua islands; in two periods, 2004–2006 and 2009–2012 were subjected for *pfdhfr* and *pfdhps* sequence analysis.

**Results:**

Different genotype polymorphisms of *pfdhfr* and *pfdhps* were observed in the parasites from various regions in Indonesia and relatively more divergent genotypes were determined from Kalimantan isolates in both 2004–2006 and 2009–2012. The parasites containing K540**T** mutation were identified in 2004–2006 isolates from East Kalimantan, East Java and Sumbawa as an S**GTG**A haplotype. The other I588**F** mutation was also determined in 2004–2006 parasites, isolated from Lombok and Sumbawa islands as an S**GE**AA(588**F**) haplotype. The parasites with *pfdhfr*/*pfdhps* quintuple or sextuple mutation, a genotype marker of SP resistance, were determined mostly in Kalimantan in both 2004–2006 and 2009–2012.

**Conclusion:**

Analysis of the prevalence and *pfdhfr/pfdhps* combined genotypes of K540**T** or I588**F** mutations suggested that K540**T** might be origin in Kalimantan Island and I588**F** in Sumbawa Island and then these were spread to other areas along with people movement. This research indicates regular monitoring of drug efficacy and parasite genotype analysis is important to keep efficiency and prevent the spread of resistance. It is also essential for the latest anti-malarial drug artemisinin-based combination therapy.

## Background

Malaria incidence in Indonesia has decreased threefold in the last decade [1], and there is no evidence for the presence of the parasites resistant to artemisinin-based combination therapy (ACT) [2]. However, about 25% of Indonesia’s population (total population: 261 million) is at risk of malaria, and more than 200,000 positive cases were reported in 2016 [3]. Detection of drug resistant malaria parasites and prevention of these spreading are critical to keep efficacy of the malaria treatment and to obtain elimination of malaria. In this research, origin and distribution of unique mutations of *Plasmodium falciparum* sulfadoxine–pyrimethamine (SP) resistance in Indonesia were analysed.

SP has been widely used as an anti-malarial drug for treatment of uncomplicated malaria, and for intermittent preventive treatment in vulnerable populations, pregnant women (IPTp) and infants (IPTi) in high malaria transmission areas in Africa [4, 5]. However, emergence and spread of SP resistant *P. falciparum* has been reported worldwide [6–9]. In Indonesia, SP was recommended as a second line anti-malarial drug after chloroquine resistance had been determined in 1973, and *P. falciparum* resistance to SP was reported for the first time in Jayapura (Papua Province) in 1979 [10, 11]. Chloroquine and SP had been used in Indonesia until 2008, when the malaria treatment policy was changed. ACT, using a combination of an artemisinin derivative with another anti-malarial, such as piperaquine, lumefantrine or amodiaquine, is provided as the first line anti-malarial drug for treatment of uncomplicated malaria, and SP is not administered for malaria treatment. However, people, especially in local areas, use SP when they are suffering from malaria, or for chemoprophylaxis [12], because of some effectiveness, low cost, simple administration as a single oral dose, and fewer side effects. It is important to obtain information about SP resistance in malaria endemic areas in Indonesia.

The mutations in *P. falciparum* dihydrofolate reductase (*pfdhfr*) and dihydropteroate synthase (*pfdhps*) are responsible for pyrimethamine and sulfadoxine resistance, respectively [13–16]. Stepwise accumulation of point mutations in *pfdhfr* and *pfdhps* genes is associated with higher level of resistance to SP in vitro and in vivo [17, 18]. The amino acid substitution at position 108 serine to asparagine or threonine (S108**N**/**T**) in *pfdhfr* is essential for subsequent A16**V**, N51**I**, C59**R** and I164**L** mutations (underlined bold type indicates the mutant allele), leading to high-level of resistance to cycloguanil or pyrimethamine [19]. Similarly, a single mutation in the *pfdhps* converting alanine to glycine at amino acid position 437 (A437**G**), which is normally the first mutation under the sulfadoxine drug pressure, conferred on the parasite a fivefold higher level of drug resistance [16]. Additional mutations K540**E** and A581**G**, then S436**A**/**F** and A613**S**/**T** are associated with increasing resistance to sulfadoxine. A combination of *pfdhfr* triple (N51**I**, C59**R** and S108 **N**) and *pfdhps* double (A437**G** and K540**E**) mutations collectively form the quintuple mutations, which is strongly associated with in vitro and in vivo SP resistance [17, 20–24].

There are several reports of mutation analysis of *pfdhfr* and *pfdhps* genes from different malaria endemic areas in Indonesia. Different ratio of mutation level was reported from sample analysis of several island and district parasites. From the West Papua sample analysis obtained in 1996 to 1999 by Nagesha et al. [25], C59**R** and S108**N** mutations in *pfdhfr* and A437**G** in *pfdhps* were commonly determined in SP resistant parasites. In addition, they reported an additional K540**E** mutation in *pfdhps* was observed in more resistant level parasites. Extensive analysis of the parasite genotypes from eight malaria endemic areas were reported by Syafruddin et al. in 2005, representing a broad region of the western and eastern Indonesian archipelago [26]. Polymorphisms in *pfdhfr* gene at S108**N**/**T**, A16**V** and C59**R** were frequently identified, in which A16**V** were observed in association with S108**T** and these were differently distributed, more common among samples from eastern regions. The polymorphism in *pfdhps* was less frequent in this report; about 15% of A437**G** and less than 5% of K540**E** were detected. Among the Sumba island samples of 2007, less frequent mutations were reported by Asih et al. in 2009 [27]; about 25% parasites presented S108**N** and C59**R** mutations in *pfdhfr* and only few % of the isolates presented A437**G** mutant allele of *pfdhps*. More prevalence of mutations in *pfdhfr* and *pfdhps* was observed from a study of SP efficacy and genotype analysis in South Kalimantan in 2009–2010 [28]. More than 90% of the isolates exhibited S108**N** and C59**R** mutations in *pfdhfr* gene, in addition I164**L** substitution was detected in 30% of the parasites. The alterations in *pfdhps* were detected at the amino acid positions of A437, K540, A581 and I588 to glycine (97%), glutamine or threonine (36%, 36%), glycine (45%) and phenylalanine (23%), respectively. This result of *pfdhfr* and *pfdhps* genotypes is quite unique compared with the previous Indonesia parasites; more prevalence of mutation ratio and detection of novel mutations of *pfdhps* K540**T** and I588**F**.

In this study, *pfdhfr* and *pfdhps* sequences of *P. falciparum* from different malaria endemic regions, mostly eastern part of Indonesia were analysed to investigate polymorphisms of *pfdhfr* and *pfdhps* genotypes and predict susceptibility for sulfadoxine–pyrimethamine in malaria parasites in Indonesia, and to obtain information about distribution of the previously identified *pfdhps* K540**T** and I588**F** novel mutations.

## Methods

### Study sites and malaria patients

Malaria parasites collected from the following several endemic areas in Indonesia were analysed in this study: Sumatera (Indragiri Hilir, Merangin), Kalimantan (Paser, Seruyan, Banjar), Java (Pacitan), Lombok (West Lombok), Sumbawa (Sumbawa), Timor (Timor Tengah Selatan), Sulawesi (Gorontalo), and Papua (Jayapura) islands in 2004–2006 and in 2009–2012. Malaria patients were recruited at primary health centers, district hospitals or local field areas and written informed consent were obtained from each participant, or from caretakers if participations were under 12 years old, after explanation of the purpose of the study in local language. Finger prick blood samples were collected on a glass slide for microscopical observation and on a filter paper (Advantec, Toyo Roshi Kaisha, Ltd., Japan) for parasite DNA analysis. Thick smear blood films were stained with Giemsa (Merck, Germany) and examined microscopically for the presence of malaria parasites. Dried blood spot on a filter paper was kept in a small plastic clips prior to parasite DNA extraction. In some field studies, BinaxNOW Malaria diagnosis kit (Binax Inc, Portland, ME, USA) were used to identify *P. falciparum* malaria patients. Patients with positive malaria diagnosis results were treated with an anti-malarial drug according to national policy. The study protocol was reviewed and approved by the Ethical Committee, Faculty of Medicine, Universitas Airlangga, Surabaya, Indonesia and Institute of Tropical Medicine, Nagasaki University, Nagasaki, Japan.

### Parasite DNA analysis

Parasite DNA was extracted from the dried blood spots on filter paper by using QIAamp DNA blood mini kit (Qiagen, Netherlands) and kept at − 30 °C. *Plasmodium falciparum* samples were selected by nested PCR methodology using species specific primer sets of 18S rRNA genes described in Snounou et al. [29] and Kimura et al. [30]. *Pfdhfr* and *pfdhps* genotypes were determined by sequencing as previously described by Isozumi et al. [31]. using amplified PCR product as a template directly for sequence analysis. Alleles corresponding to amino acid positions at 16, 51, 59, 108 and 164 of the *pfdhfr* gene and at 436, 437, 540, 581, 588 and 613 of the *pfdhps* gene were read more carefully, and at least two independent PCR products were prepared for sequence analysis in the case of rare mutations. Previously reported results from South Kalimantan Province [28] were included in this analysis.

### Statistical analysis

Data were entered in Microsoft Excel and exported to SPSS version 17.0 for analysis. Chi square and Fisher’s exact tests were used, where applicable, to assess the relationship of mutations between two periods of studies. Allele proportions were calculated as the number carrying a certain allele divided by the number of samples with positive PCR outcome.

## Results

### Sample characteristics

A total of 622 *P. falciparum* samples from symptomatic to asymptomatic malaria patients during the two periods, 2004–2006 and 2009–2012, were analysed in this study. The research areas were different levels of malaria endemicity, and presented seasonal differences in number of malaria patients. Same districts in Lombok and Papua islands, the latter is reported as high malaria endemicity, were included for sample analysis in both periods. Of the 384 samples from 622 patients, *P. falciparum pfdhfr* and *pfdhps* genes were successfully amplified and analysed both genotypes (Tables 1, 2, 3). The genotypes were obtained from more than 20 samples of Kalimantan, Lombok and Papua isolates in both periods of 2004–2006 and 2009–2012, except Papua in 2004–2006 which was 9 samples.

**Table 1:** Number of cases with each *pfdhfr* genotype allele* from different districts in Indonesia in two study periods

Place			n** (mixed cases)	2004–2006			
Island	Province	District		ANCSI	ANCNI	ANRNI	ANRNL
Sumatera	Riau	Indragiri Hilir	6			6	
Kalimantan	East Kalimantan	Paser	34			29	5
			(2)			(2^a^)/2	(2^a^)/2
Java	East Java	Pacitan	29			19	10
Lombok	West Nusa Tenggara	West Lombok	45			45	
Sumbawa	West Nusa Tenggara	Sumbawa	78	1	5	72	
			(2)	(2^a^)/2		(2^a^)/2	
Papua	Papua	Jayapura	9			9	
			201	1	5	180	15
			(4)	(2)/2		(4)/2	(2)/2

Place			n** (mixed cases)	2009–2012			
Island	Province	District		ANCSI	ANCNI	ANRNI	ANRNL
Sumatera	Riau	Indragiri Hilir	1			1	
Sumatera	Jambi	Merangin	1				1
Kalimantan	Middle Kalimantan	Seruyan	19			1	18
Kalimantan	South Kalimantan	Banjar	58		2	36	20
			(1)		(1^a^)/2	(1^a^)/2	
Lombok	West Nusa Tenggara	West Lombok	48			48	
Timor	East Nusa Tenggara	Timor Tengah Selatan	3			3	
Sulawesi	Gorontalo	Gorontalo	2			2	
Papua	Papua	Jayapura	46	5		41	
			178	5	2	132	39
			(1)		(1)/2	(1)/2	

* pfdhfr genotype alleles are presented with the amino acids at the position of 16, 51, 59, 108, and 164. The underlined bold type indicates amino acid substitution

** Number of mixed infection cases are presented in brackets as (N), and each pair of the mixed case is presented with superscript as (N^a^)/2

**Table 2:** Number of cases in each *pfdhps* genotype from different districts in Indonesia in two study periods

Place			n (mixed cases)	2004–2006						
Island	Province	District		SAKAA	SGKAA	SGKGA	SGEAA	SGEAA	SGEGA	SGTGA
								(588F)		
Sumatera	Riau	Indragiri Hilir	6	6						
Kalimantan	East Kalimantan	Paser	27			1	4			22
			(9)				(6^a^+3^b^)/2		(6^a^)/2	(3^b^)/2
Java	East Java	Pacitan	29			5	4		1	19
Lombok	West Nusa Tenggara	West Lombok	44	41				3		
			(1)	(1^a^)/2			(1^a^)/2			
Sumbawa	West Nusa Tenggara	Sumbawa	77	49	8		6	12		2
			(3)	(2^a^+1^b^)/2	(2^a^)/2		(1^b^)/2			
Papua	Papua	Jayapura	9				9			
			192	96	8	6	23	15	1	43
			(13)	(4)/2	(2)/2		(11)/2		(6)/2	(3)/2

Place			n (mixed cases)	2009–2012						
Island	Province	District		SAKAA	SGKAA	SGKGA	SGEAA	SGEAA	SGEGA	SGTGA
								(588F)		
Sumatera	Riau	Indragiri Hilir	1		1					
Sumatera	Jambi	Merangin	1						1	
Kalimantan	Middle Kalimantan	Seruyan	19			11	6		1	1
Kalimantan	South Kalimantan	Banjar	56	1	10	4	6	8		27
			(3)			(2^a^)/2	(1^b^)/2	(2^a^)/2		(1^b^)/2
Lombok	West Nusa Tenggara	West Lombok	48	47			1			
Timor	East Nusa Tenggara	Timor Tengah Selatan	3	3						
Sulawesi	Gorontalo	Gorontalo	2		1			1		
Papua	Papua	Jayapura	46	17			25	4		
			176	68	12	15	38	13	2	28
			(3)			(2)/2	(1)/2	(2)/2		(1)/2

* pfdhps genotype alleles are presented with the amino acids at the position of 436, 437, 540, 581, 613 (and 588). The underlined bold type indicates amino acid substitution

** Number of mixed infection cases are presented in brackets as (N), and each pair of the mixed case is presented with superscript as (N^a^)/2

**Table 3:** Number of cases with each pfdhfr*/pfdhps** combined genotype* from different districts in Indonesia in two study periods

Year	Island	Province	District	n*** (mixed cases)	*Pfdhfr/pfdhps* combined genotypes							
					ANCSI*		ANC**N**I		AN**RN**I			
					SAKAA**	S**GE**AA	SAKAA	S**G**K**G**A	SAKAA	S**G**KAA	S**G**K**G**A	S**GE**AA
2004–2006	Sumatera	Riau	Indragiri Hilir	6					6			
	Kalimantan	East Kalimantan	Paser	25							1	4
				(11)								(6^a^+3^b^)/2
	Java	East Java	Pacitan	29								
	Lombok	West Nusa Tenggara	West Lombok	44					41			
				(1)					(1^a^)/2			(1^a^)/2
	Sumbawa	West Nusa Tenggara	Sumbawa	77	1		5		43	8		6
				(3)	(2^a^)/2				(1^b^)/2	(2^a^)/2		(1^b^)/2
	Papua	Papua	Jayapura	9								9
				190	1		5		90	8	1	19
				(15)	(2)/2				(2)/2	(2)/2		(1)/2

Year	Island	Province	District	n*** (mixed cases)	*Pfdhfr/pfdhps* combined genotypes							
					AN**RN**I			AN**RNL**				
					S**GE**AA (588**F**)	S**GEG**A	S**GTG**A	S**G**KAA	S**G**K**G**A	S**GE**AA	S**GEG**A	S**GTG**A
2004–2006	Sumatera	Riau	Indragiri Hilir	6								
	Kalimantan	East Kalimantan	Paser	25			15					5
				(11)		(6^a^)/2	(3^b^+2^c^)/2					(2^c^)/2
	Java	East Java	Pacitan	29			19		5	4	1	
	Lombok	West Nusa Tenggara	West Lombok	44	3							
				(1)	12		2					
	Sumbawa	West Nusa Tenggara	Sumbawa	77								
				(3)								
	Papua	Papua	Jayapura	9								
				190	15		36		5	4	1	5
				(15)		(6)/2	(5)/2					(2)/2

Year	Island	Province	District	n*** (mixed cases)	*Pfdhfr/pfdhps* combined genotypes							
					ANCSI*		ANC**N**I		AN**RN**I			
					SAKAA**	S**GE**AA	SAKAA	S**G**K**G**A	SAKAA	S**G**KAA	S**G**K**G**A	S**GE**AA
2009–2012	Sumatera	Riau	Indragiri Hilir	1						1		
	Sumatera	Jambi	Merangin	1								
	Kalimantan	Middle Kalimantan	Seruyan	19								
	Kalimantan	South Kalimantan	Banjar	56			1	1			4	5
				(3)				(1^a^)/2			(1^b^)/2	
	Lombok	West Nusa Tenggara	West Lombok	48					47			1
	Timor	East Nusa Tenggara	Timor Tengah Selatan	3					3			
	Sulawesi	Gorontalo	Gorontalo	2						1		
	Papua	Papua	Jayapura	46	1	4			16			21
				176	1	4	1	1	66	2	4	27
				(3)				(1)/2			(1)/2	

Year	Island	Province	District	n*** (mixed cases)	*Pfdhfr/pfdhps* combined genotypes							
					AN**RN**I			AN**RNL**				
					S**GE**AA (588**F**)	S**GEG**A	S**GTG**A	S**G**KAA	S**G**K**G**A	S**GE**AA	S**GEG**A	S**GTG**A
2009–2012	Sumatera	Riau	Indragiri Hilir	1								
	Sumatera	Jambi	Merangin	1							1	
	Kalimantan	Middle Kalimantan	Seruyan	19			1		11	6	1	
	Kalimantan	South Kalimantan	Banjar	56	8	1	17	10				9
				(3)	(1^a^+1^b^)/2					(1^c^)/2		(1^c^)/2
	Lombok	West Nusa Tenggara	West Lombok	48								
	Timor	East Nusa Tenggara	Timor Tengah Selatan	3								
	Sulawesi	Gorontalo	Gorontalo	2	1							
	Papua	Papua	Jayapura	46	4							
				176	13	1	18	10	11	6	2	9
				(3)	(2)/2					(1)/2		(1)/2

* *pfdhfr* genotype are presented with the amino acids at the position of 16, 51, 59, 108, 164, and ANCSI is the wild type of *pfdhfr*

** *pfdhps* genotype are presented with the amino acids at the position of 436, 437, 540, 581, 613 (and 588), and SAKAA is the wild type of *pfdhps*. The underlined letters indicate amino acid substitutions

*** Number of mixed infection cases are presented in brackets as (N), and each pair of the mixed case is presented with superscript as (N^a^)/2

### *Pfdhfr* and *pfdhps* genotypes

**Fig. 1:**
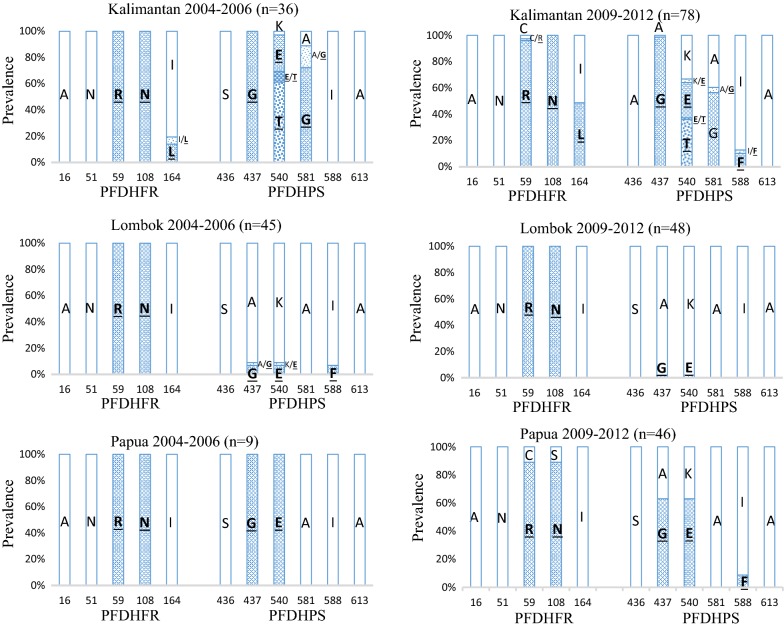
Prevalence of *pfdhfr* and *pfdhps* mutations in *Plasmodium falciparum* isolates from Kalimantan, Lombok and Papua patients in 2004–2006 and 2009–2012. The horizontal axis is the amino acid position or codon of mutations in *pfdhfr* and *pfdhps* genes. The white box indicates the wild-type and the texture boxes are mutants written by amino acid abbreviations. A = alanine, N = asparagine, R = arginine, I = isoleucine, L = leucine, S = serine, G = glycine, K = lysine, E = glutamic acid, T = threonine, F = phenylalanine, C = cysteine. Mixed genotype infections are presented as I/**L** or **E**/**T** et al.

The *pfdhfr* and *pfdhps* genotypes were determined from 384 samples, 205 in 2004–2006 and 179 in 2009–2012. Polymorphisms were identified at amino acid positions of C59, S108 and I164 in *pfdhfr* to arginine, aspargine and leucine, respectively, but no other substitutions including at A16 and N51 were observed. In *pfdhps* gene, substitutions were observed at amino acid positions of A437, K540, A581 and I588 to glycine, glutamic acid or threonine, glycine and phenylalanine, respectively. No mutations were detected at the positions of S436 and A613. Comparison of the mutation ratios in 2004–2006 and 2009–2012 samples from Kalimantan, Lombok and Papua is presented in Fig. 1. Similar ratio of mutation was observed in 2004–2006 and 2009–2012 at each amino acid position of *pfdhfr* in Kalimantan, Lombok and Papua samples, but more mutation ratio was observed at I164**L** in 2009–2012 than 2004–2006 in Kalimantan. Some different percentages of the mutations were detected in *pfdhps* gene from Kalimantan, Lombok and Papua samples, and between 2004–2006 and 2009–2012 in the same island parasite samples. Remarkable change was noticed in the *pfdhps* I588**F** mutation; new appearance in Kalimantan and Papua samples in 2009–2012 isolates, but disappearance from Lombok samples in 2009–2012.

With the *pfdhfr* mutations at C59**R**, S108**N** and I164**L**, in total, the wild type *pfdhfr* allele ANCSI (at positions 16, 51, 59, 108 and 164, respectively) and three mutant alleles, ANC**N**I, AN**RN**I and AN**RNL** were detected in this study (Table 1).

Relatively high ratio of mutation in *pfdhps* gene was determined at A437 to glycine (Fig. 1), which is regarded as the first and most essential substitution for sulfadoxine resistance [16]. Two types of mutations at amino acid position K540 were detected as AAA (lysine codon) to GAA (glutamic acid) or ACA (threonine). One of this K540**T** and another unique mutation I588**F** are recently reported alleles in Indonesia, recognized through previous South Kalimantan sample analysis [28]. The K540**T** was also reported in Sabah, Malaysia [32], which is located in the same island. Altogether, the wild type *pfdhps* haplotype SAKAA (at positions 436, 437, 540, 581 and 613, respectively) and six different mutant alleles S**G**KAA, S**G**K**G**A, S**GE**AA, S**GE**AA(588**F**), S**GTG**A and S**GEG**A were identified in this study (Table 2). The unique I588**F** mutation was detected only in S**GE**AA(588**F**) haplotype and K540**T** was only in S**GTG**A allele in both 2004–2006 and 2009–2012 samples.

### Diverse polymorphisms in different malaria endemic areas

**Fig. 2:**
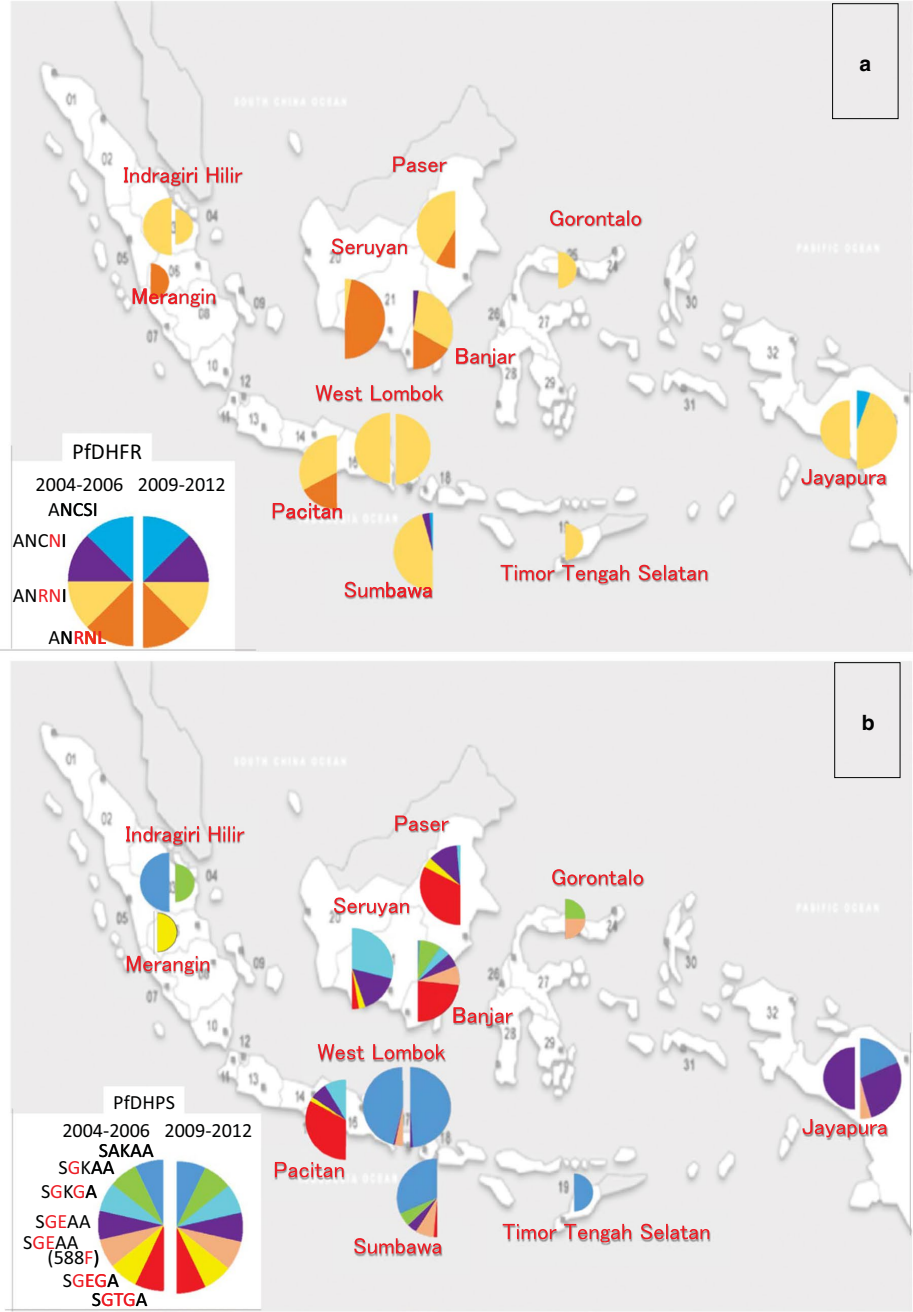
Distribution of *pfdhfr* (**a**) and *pfdhps* (**b**) haplotypes in Indonesia. Genotype polymorphism in 2004–2006 parasites are shown in left half semicircles and 2009–2012 parasites in right semicircles. The various *pfdhfr* or *pfdhps* alleles are presented with different colours. Larger, middle and small semicircles represent number of parasites analysed, more than 10, 5 to 9 and less than 4 samples, respectively

Among the observed four *pfdhfr* genotypes with simple stepwise accumulation pattern of mutations, AN**RN**I is the most common *pfdhfr* allele in most of the research locations both in 2004–2006 and 2009–2012 periods except Seruyan (Middle Kalimantan) in 2009–2012, where the AN**RNL** is dominant (Fig. 2a). The AN**RNL** allele is frequently detected in Paser (East Kalimantan) and Pacitan (East Jawa) in 2004–2006 samples, and Banjar (South Kalimantan) in 2009–2012. A remarkable observation is the wild type ANCSI allele found in Jayapura (Papua) samples in 2009–2012 had not been observed in 2004–2006.

Complicated genotype variations were observed in *pfdhps* allele haplotypes. Similar variant haplotypes were detected from Paser (East Kalimantan) and Pacitan (Easr Java) in 2004–2006 samples, but different haplotype variations were observed in parasite populations from most of the research areas (Fig. 2b). The substitution of K540**E** was detected as one of the S**GE**AA, S**GE**AA(588**F**) or S**GEG**A alleles at most of the malaria research areas in 2004–2006 and 2009–2012, except Indragiri Hilir (Riau, Sumatera Island), and Timor Tengah Selatan (Timor Island), where only small number of samples were obtained (Table 2). The unique I588**F** substitution was detected from the parasites with K540**E** mutation as the *pfdhps* S**GE**AA(588**F**) allele only in Lombok and Sumbawa Island samples in 2004–2006. It would have been then spreading to the other islands of eastern Indonesia and detected from Banjar in Kalimantan, Gorontalo in Sulawesi and Jayapura in Papua Islands in 2009–2012 (Fig. 2b). This mutant was not detected in Lombok in 2009–2012.

Another unique mutation of K540**T** was identified as a major *pfdhps* S**GTG**A allele in Paser (East Kalimantan) and Pacitan (East Java), and in Sumbawa as a minority in 2004–2006 (Fig. 2b). This was then detected only in Kalimantan Island in 2009–2012, as a major allele of Banjar (South Kalimantan) parasites, and one case of Seruyan (Middle Kalimantan) isolates (Fig. 2b).

### Combined genotypes of *pfdhfr*/*pfdhps* haplotypes

With a combination of four *pfdhfr* and seven *pfdhps* haplotypes, totally 16 different combined *pfdhfr*/*pfdhps* genotypes were determined; 13 combined genotypes in 2004–2006 parasites and 16 in 2009–2012 samples (Table 3). Relatively more polymorphisms in the *pfdhfr*/*pfdhps* combined genotype were observed in Paser (East Kalimantan), Pacitan (East Java), Sumbawa samples in 2004–2006, and Seruyan (Middle Kalimantan), Banjar (South Kalimantan) and Jayapura (Papua) samples in 2009–2012. Only one AN**RN**I/S**GE**AA genotype was observed in 2004–2006 Jayapura samples, and several different combined genotypes were detected in 2009–2012 samples. The originally detected AN**RN**I/S**GE**AA remained a major genotype, but introduction of the parasites with the wild type *pfdhfr* and *pfdhps* between two research periods had increased number of types in the combined genotype.

**Fig. 3:**
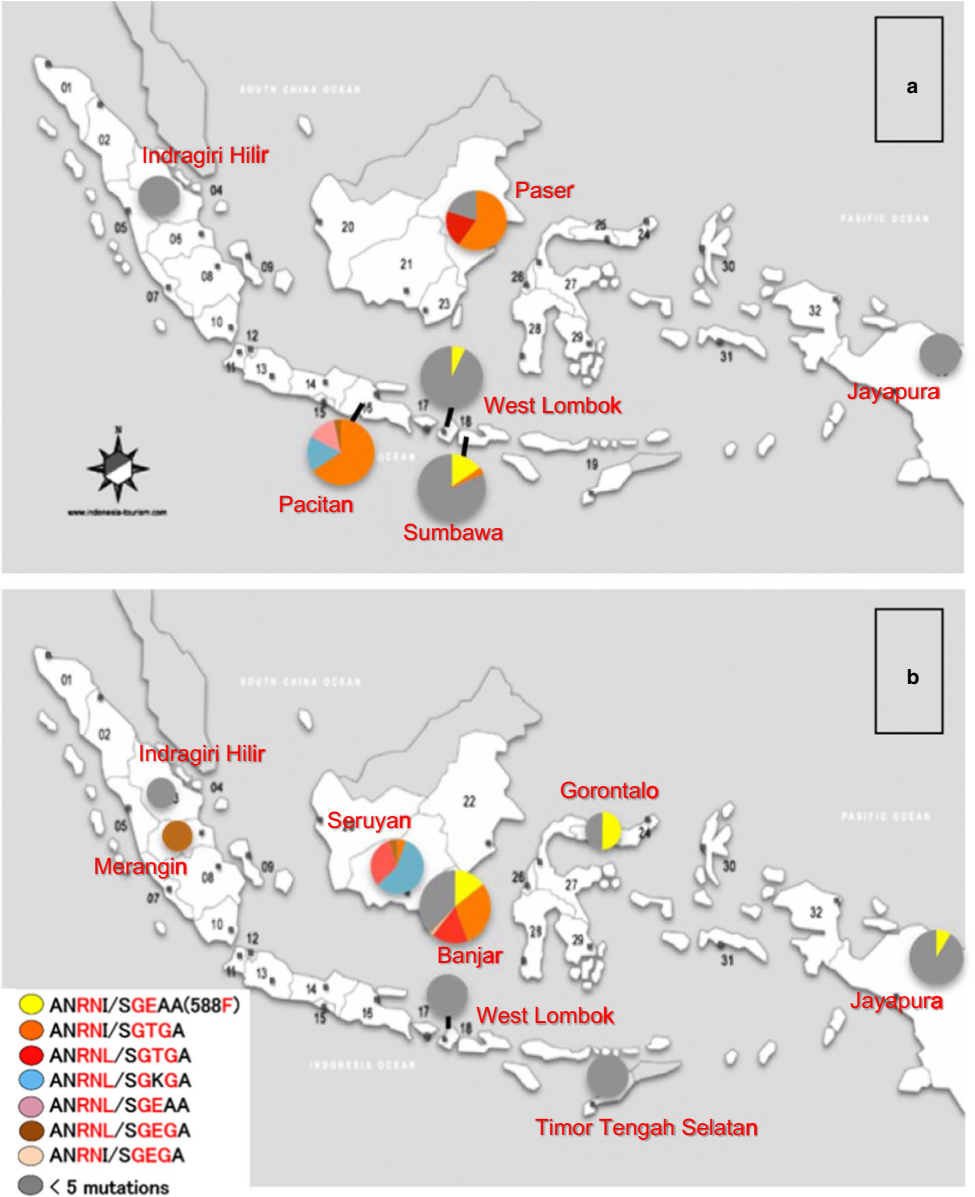
Distribution of *pfdhfr*/*pfdhps* combined quintuple and sextuple mutant haplotypes in 2004–2006 (**a**) and 2009–2012 (**b**). In this case, *pfdhps* 588**F** is included for counting number of mutation and AN**RN**I/S**GE**AA(588**F**) is involved as the quintuple mutant

Accumulation of mutations in *pfdhfr* and *pfdhps* genes enhances parasite resistance level against sulfadoxine–pyrimethamine. The parasites containing more than five mutations in combined *pfdhfr*/*pfdhps* genotype have been shown not to respond adequately to SP treatment [8, 22–24]. Prevalence of the quadruple AN**RN**I/S**GE**AA(588**F**) genotype, and quintuple or sextuple mutant genotypes in each research area is presented in Fig. 3. The parasites from Paser (East Kalimantan) and Pacitan (East Java) in 2004–2006 and from Seruyan (Middle Kalimantan) and Banjar (South Kalimantan) in 2009–2012 mostly presented SP resistant genotypes, especially the Pacitan parasite samples in 2004–2006 and Seruyan parasites in 2009–2012 were all belonging to the quintuple or sextuple mutants. The parasites from the other areas presented the genotypes containing four or less mutations in combination genotype that tend to respond adequately to SP treatment. Prevalence of parasite ratios harboring five or more mutations between 2004–2006 and 2009–2012 are not significantly different between two periods of time in Kalimantan, Lombok and Papua statistically (Table 4).

**Table 4:** Prevalence of the parasites with *pfdhfr/pfdhps* quintuple and sextuple mutation in 2004–2006 and 2009–2012

Number of mutation*	2004–2006		2009–2012		*P*-value**
	n	%	n	%	
	Kalimantan (n = 36)		Kalimantan (n = 78)		
≥ 5	31	86.1	57	73.1	0.153
< 5	5	13.9	21	26.9	
	Lombok (n = 45)		Lombok (n = 48)		
≥ 5	3	6.7	0	0	0.109
< 5	42	93.3	48	100	
	Papua (n = 9)		Papua (n = 46)		
≥ 5	0	0	4	8.7	1
< 5	9	100	42	91.3	

* Quintuple, sextuple mutants are parasites containing five or six mutations in the *pfdhfr*/*pfdhps* combined haplotype. In this case, *pfdhps* 588**F** is included for counting number of mutation

** P-values were calculated by comparing actual number of patients presenting each genotype by Fisher’s Exact test

## Discussion

In this report, polymorphisms of *pfdhfr* and *pfdhps* genes in several malaria endemic areas in Indonesia are analysed in two periods of time, 2004–2006 and 2009–2012. Previously reported amino acid substitutions, important for sulfadoxine–pyrimethamine (SP) resistance in Indonesian parasites, were determined in this analysis. The C59**R**, S108**N** and I164**L** mutations in *pfdhfr* gene and A437**G**, K540**E**/**T**, A581**G** and I588**F** in *pfdhps* were observed from both 2004–2006 and 2009–2012 samples, but *pfdhfr* A16**V** and S108**T** mutations for cycloguanil resistance were not detected (Fig. 1). In *pfdhfr* gene, wild type ANCSI and three mutant ANC**N**I, AN**RN**I and AN**RNL** alleles were observed (Table 1). This simple accumulation pattern of mutations in the *pfdhfr* genotype supports the stepwise selection hypothesis of resistant gene evolution [19]. Wild type SAKAA and six different mutant alleles were determined in *pfdhps* gene (Table 2). Comparison of wild type and each mutant alleles suggests stepwise accumulation of mutations in the *pfdhps* genotypes.

The unique K540**T** and I588**F** mutations of *pfdhps*, both of which were detected previously from 2009 to 2010 South Kalimantan sample analysis [28] and the former was also reported by Lau et al. [32] from 2010 Sabah, Malaysia parasites, were identified in 2004–2006 parasites. The evidence presenting here that K540**T** was detected in 2004–2006 as an S**GTG**A haplotype from several parasites in East Kalimantan, East Java and Sumbawa Island suggests a possibility of Kalimantan Island origin for this mutation. In East Kalimantan, the S**GTG**A was found from many patients as a combination genotype of either AN**RN**I/S**GTG**A or AN**RNL**/S**GTG**A, additionally as a mixed infection of both genotypes (Table 3). Meanwhile, the S**GTG**A existed as the combination genotype of AN**RN**I/S**GTG**A only in East Java and Sumbawa parasites.

Another novel mutation I588**F** was also detected in 2004–2006 samples from Lombok and Sumbawa islands; the parasites of S**GE**AA both with and without I588**F** mutation were detected in Sumbawa, whereas S**GE**AA(588**F**) and wild type SAKAA were observed in Lombok. It suggests a possibility that the initial mutation of I588**F** had occurred in S**GE**AA type parasites in Sumbawa Island, then introduced into its neighbor Lombok Island. Further analysis and comparison of microsatellite loci around the *pfdhps* haplotypes will provide additional information for understanding the origin and spreading of these K540**T** and I588**F** mutant alleles.

In Riau and Jambi Provinces (Sumatera Island), only small number of malaria patients had been detected and these were mostly infected with *P. vivax* and only a few *P. falciparum* were observed in both 2004–2006 and 2009–2012 periods. Timor Tengah Selatan (Timor Island) and Gorontalo (Sulawesi Island) were similar situation in 2009–2012. Remarkably *pfdhps* I588**F** mutation was observed in Gorontalo as the same combined genotype of AN**RN**I/S**GE**AA(588**F**).

Genotype comparison of 2004–2006 and 2009–2012 parasites in each malaria areas provides additional information (Table 3). In Lombok, parasites of the major combined genotype AN**RN**I/SAKAA and a few number of minor type were detected in both 2004–2006 and 2009–2012 periods. The parasites might have well responded to SP or other malaria therapies and new SP resistant type parasites had not settled in Lombok. On the other hand, increased number of *pfdhfr*/*pfdhps* combined genotype was determined in 2009–2012 parasites from Papua Island compared to 2004–2006 parasites. The genotypes were AN**RN**I/S**GE**AA(588**F**) and new combinations with either *pfdhfr* or *pfdhps* wild type. This suggests some parasites with wild type *pfdhfr* ANCSI and *pfdhps* SAKAA were introduced as the both wild type combination or either haplotype individually between two periods. The AN**RN**I/S**GE**AA had been selected under SP pressure before 2004–2006, and the new parasites with *pfdhfr* and/or *pfdhps* wild type alleles and AN**RN**I/S**GE**AA(588**F**) were maintained under reduced SP pressure after DHP was commonly used. ACT was introduced in Papua Province in 2005; first artesunate-amodiaquine (AA) was used experimentally, then changed to dihydroartemisinin–piperaquine (DHP) in 2008 [33]. Wild type *pfdhfr* and *pfdhps* genotype detection was reported in the parasites from West Papua and several islands by Syafruddin et al. in 2005 [26]. It suggests the parasites with wild type allele were introduced from a neighbour districts or it might exist in Papua in 2004–2006, however it could not detected because the number of samples was not sufficient.

Parasite populations from different Provinces in Kalimantan Island presented diverse polymorphisms. Analysis of Paser (East Kalimantan) 2004–2006 parasites revealed dominance of the *pfdhps* K540**T** mutation (S**GTG**A haplotype, Fig. 2b) and many cases of mixed genotype infections (Table 3). This suggests high *P. falciparum* infection rate under strong pressure of SP. The mixed genotype infection was not common in 2009–2012 samples from Middle and South Kalimantan, and the parasites from these Provinces presented characteristic genotype polymorphisms. Most of the parasites from Seruyan (Middle Kalimantan) acquired *pfdhfr* I164**L** mutation (AN**RNL** haplotype, Fig. 2a), and several *pfdhps* genotypes involving unique *pfdhps* K540**T** or I588**F** mutations were detected in Banjar (South Kalimantan) parasites (Fig. 2b).

High heterogeneity of malaria epidemiology and divergent genetic polymorphisms across islands, districts, and even close neighbour sub-districts in one island are not uncommon in Indonesia [26]. Divergent polymorphisms in *pfdhfr*/*pfdhps* genotype of the parasite populations from different Kalimantan provinces were observed in this study from analysis of the parasite samples before introduction of ACT at each research site in these Provinces. AA was implemented in 2006 in East Kalimantan and then DHP treatment was started in 2009. DHP has been first-line treatment therapy against malaria in Middle Kalimantan and South Kalimantan since 2010. However in fact, ACT was introduced gradually form one site to the other, and the parasite samples from Kalimantan analysed in this research were collected before ACT was applied in the research areas. Current situation and comparison of genetic divergence among Kalimantan provinces are important subject to provide information how application of ACT influences malaria parasite populations on genotypes and polymorphisms.

Different prevalence of quintuple or sextuple mutant parasites in *pfdhfr*/*pfdhps* combined genotypes were observed (Fig. 3). While the parasites from Kalimantan and Pacitan (East Java) belonged to SP resistant quintuple or sextuple mutant genotype, parasites from the other areas in Indonesia presented four or less mutations in combined genotype that tend to adequate response for SP treatment. In addition, efficacy of SP treatment for *P. vivax* malaria infection in Indonesia was reported by Asih et al. [34] recently. These suggest SP could be considered for prevention or treatment of malaria as a single prescription or in combination with artemisinin in Indonesia except in Kalimantan Island. In such a case, regular monitoring of the efficacy and regular genotype analysis are essential to prevent spread of resistance.

Mobility of people among thousands of islands is one of the important factors for malaria control in Indonesia. Some of outbreaks, resurgences of malaria had occurred under the unique circumstances of people migration. Marwoto et al. reported immigrant workers who worked as transmigrants or seasonal workers in malaria endemic areas outside Java Island returned to their home villages brought imported malaria cases [35, 36]. Many migrants and temporal workers have moved from Pacitan (East Java) to several islands historically. Similar *pfdhfr*/*pfdhps* genotype polymorphisms in 2004–2006 parasites from Pacitan (East Java) and 2009–2012 Saruyan (Middle Kalimantan) in Table 3 suggests the malaria parasites in these districts could have been transferred along with human migrations.

The Indonesia National Malaria Control Programme desires to eliminate malaria in the whole country by 2030 [37]. Most districts in Java and Bali islands, also several districts in other islands fall under the World Health Organization criteria of elimination and malaria cases have gradually decreased over the last several years in Indonesia [38].

Considering presence of malaria vector mosquitoes in most of the places used to be malaria endemic, and current situation of frequent human migrations within the country, continuous maintenance of early malaria diagnosis and treatment system is essential for malaria elimination programme. Parasite genotype analysis is helpful to follow malaria transmission and drug treatment efficiencies.

## Conclusion

Different polymorphisms of *pfdhfr*/*pfdhps* genotypes from malaria endemic areas in Indonesia were observed in both 2004–2006 and 2009–2012 parasite isolates. The unique K540**T** mutations of *pfdhps* were detected as an S**GTG**A haplotype in the parasites from Kalimantan and Sumbawa islands, and East Java. The other *pfdhps* I588**F** mutation were observed as an S**GE**AA(588**F**) haplotype in Sumbawa and Lombok parasites in 2004–2006 and South Kalimantan, Gorontalo (Sulawesi) and Papua in 2009–2012. Analytical study of the prevalence and *pfdhfr/pfdhps* combined genotypes of K540**T** or I588**F** mutations suggested that K540**T** might be origin in Kalimantan Island and I588**F** in Sumbawa Island and then these were brought to other areas together with people movement. This research indicates regular monitoring of drug efficacy and parasite genotype analysis is important to keep efficiency and prevent the spread of resistance. It is also essential for the latest anti-malarial drug artemisinin-based combination therapy (ACT).
